# Pregnancy Outcomes among Patients with Sickle Cell Disease in Brazzaville

**DOI:** 10.1155/2020/1989134

**Published:** 2020-09-15

**Authors:** F. O. Galiba Atipo Tsiba, C. Itoua, C. Ehourossika, N. Y. Ngakegni, G. Buambo, N. S. B. Potokoue Mpia, A. Elira Dokekias

**Affiliations:** ^1^Hematology Department, University Hospital of Brazzaville (Congo), Brazzaville, Congo; ^2^Faculty of Health Sciences, Marien Ngouabi University of Brazzaville (Congo), Brazzaville, Congo; ^3^Gynecology-Obstetrics Department, University Hospital of Brazzaville (Congo), Brazzaville, Congo; ^4^Pediatric Department, University Hospital of Brazzaville (Congo), Brazzaville, Congo

## Abstract

**Introduction:**

Sickle cell disease (SCD) is one of the most common genetic diseases in the world. It combines, in its homozygous form, chronic hemolytic anemia, vasoocclusive complications, and susceptibility to infections. It is well known that the combination of pregnancy and sickle cell disease promotes the occurrence of complications that are sometimes fatal for the mother and/or the fetus.

**Objective:**

The objective of the current study was to compare pregnancy outcomes among women with SCD with those of women without the diagnosis of SCD. *Materials and methods*. It was a case-control study carried out in four maternity hospitals in Brazzaville in 2 years (July 2017–June 2019). It concerned 65 parturients with SS homozygous SCD. The mode of childbirth and maternal and perinatal morbidity and mortality were compared with those of 130 non-sickle cell pregnant women.

**Results:**

The average age was 27 years for SCD women and 31 years for non-SCD women. The average gestational age at delivery was 35 weeks for SCD women and 38 weeks for non-SCD women. From the logistic regression analysis using the comparison group as the reference group, there was excessive risk in SCD compared to non-SCD of infection (29.3% vs. 4.6%, OR = 21.7, 95% CI [7.6–62.7]; *p*=0.001), cesarean (63% vs. 35.4%, OR = 3.1, 95% CI [1.6–5.7]; *p*=0.001), prematurity (75.4% vs. 30.8%, OR = 8, 95% CI [3.0–23.2]; *p*=0.001), low birth weight (52.3% vs. 16.1%, OR = 4.7, 95% CI [2.4–9.4]; *p*=0.001), neonatal requiring admission to the intensive care unit (40.3% vs. 17.5%, OR = 3.2, 95% CI [1.6–6.3]; *p*=0.01), and neonatal death (21.5% vs. 4.8%, OR = 4.3, 95% CI [1.5–12.2]; *p*=0.01).

**Conclusion:**

The risk of pregnancy in patients with homozygous sickle cell anemia remains high, on both the maternal and fetal sides.

## 1. Introduction

The substitution of glutamic acid by valine in position 6 in the *β*-globin chain characterizes the abnormal hemoglobin (HbS) of sickle cell anemia. This results in the fragility of the red blood cell which deforms when the oxygen pressure decreases [1]. Its homozygous form combines anemia, vasoocclusive complications, and susceptibility to infections. In sub-Saharan Africa, where the majority of patients are found, the disease is often fatal before the age of 5 [2]. The improvement in survival secondary to therapeutic progress allows more and more pregnancies in sickle cell women [3]. However, these pregnancies are at very high maternal and fetal risk [4]. In Congo Brazzaville, a study carried out over 25 years ago reported alarming results with high rates of maternal and fetal mortality [5]. Since then, the prognosis of these patients seems to have improved considerably, as shown by a more recent study [6]. However, the latter has limitations, in particular, its descriptive and especially monocentric nature. The objective of this work was to describe the mode of delivery and the outcome of pregnancy in homozygous sickle cell patients in the four main maternity hospitals in Brazzaville.

## 2. Materials and Methods

This was a case-control study carried out in the obstetrics and gynecology departments of the 4 main hospitals in the city of Brazzaville: University Hospital of Brazzaville (UHB), Specialized Mother and Child Hospital Blanche Gomez (SMCH), Talangaï Hospital (TH), and Makélékélé Hospital (MH). It was conducted over a period of 24 months (July 01, 2017–June 30, 2019). The case group was made up of pregnant women with SS homozygous sickle cell disease. The control group was made up of non-SCD gestants (Hb AA), two controls for one case. Excluded were women whose delivery time was less than 28 weeks of amenorrhea (in our country, the resuscitation of children born before 28 weeks of gestation is extremely delicate, and we therefore chose this age limit to assess the fetal prognosis), women admitted to the expulsion phase, or women who gave birth outside the centers mentioned above.

The minimum sample size was calculated according to the Schlesselman formula taking into account the data from the Muganyizi study in Tanzania [3].

The parameters analyzed were as follows:

- Maternal: age, complications linked to sickle cell disease
- Obstetric: gestational age (prematurity before 37 weeks of gestation), mode of delivery, postpartum complications
- Fetal: birth weight (low birth weight was defined as a birth weight <2500 g), complications requiring admission to the intensive care unit, mortality.

The chi-square test was used for the comparison of the proportions, the Student *t*-test for that of the means, and the Mann–Whitney for that of the medians. The odds ratio with the 95% confidence interval was calculated to assess the association between two variables. The *p* value of the probability was considered significant for a value < 0.05. Logistic regression was applied in order to identify the associated factors having a great power and to eliminate those of confusion.

## 3. Results

During the study period, there were 80 deliveries of pregnant SCD women. Sixty-five of them were included in the study and their data had been compared to that of 130 non-SCD parturients. The average age was 27 years (17 years–42 years) for SCD women and 31 years (16 years–41 years) for non-SCD women. The average gestational age at delivery was 35 weeks (34 weeks–37 weeks) for SCD women and 38 weeks (37 weeks–39 weeks) for non-SCD women.

Among SCD women, thirty-seven of them were followed either by a hematologist (89.2%) or by a pediatrician (10.8%). Twenty-seven of these had received prophylactic blood transfusions either simple or in the form of a transfusion exchange. On admission, the average hemoglobin level was 7.5 g/dL (5.5 g/dL–11 g/dL).

Table 1 shows the distribution of pregnant women according to their reproductive characteristics. During the prenatal period, infections were found in 25 women (76% vs. 24%, *p* < 0.05). The overall term delivery rate was 54.3%, with a significant difference between the two groups. Prematurity was more present in sickle cell anemia (75.4% vs. 24.6%, *p* < 0.05). Cesarean was performed in 85 women.  Table 2 shows the distribution between cases and controls. This same table also shows the other parameters analyzed and compared between the women of the two groups.

## 4. Discussion

It is well known that pregnancy and sickle cell disease have reciprocal influences on each other and that their association is a risky situation. The maternal and fetal complications observed in our study are those conventionally found in the literature [3, 7–14]. In sickle cell anemia, the risk of infection is significantly higher during pregnancy [1].

Our study did not reveal any statistical link between sickle cell anemia and maternal death. According to studies, the risk is multiplied by 19, 29, or even 117 compared to a control population [3, 4, 15]. The causes are variable: infections, embolism, eclampsia, acute chest syndrome, or multivisceral failure [3, 4, 14, 15].

Homozygous sickle cell disease is associated with a risk of low birth weight 4 times higher than in the general population [4]. The cause is chronic hypoxia of the fetal-placental unit, which itself is the consequence of anemia and rheological anomalies in the placental level [16–18]. Our results are comparable to those of the literature.

The risk of preterm delivery is very high in sickle cell women with reported rates between 9% and 48%. Several etiologies can be advanced to explain these rates. The infection encountered during pregnancy is a recognized factor in “spontaneous” prematurity. In addition, the frequency of fetal pathologies is at the origin of the medical induction of preterm birth [3, 8, 9, 11–14]. A meta-analysis found twice the risk for patients with sickle cell disease SS than for controls [4]. Its causes deserve to be studied in our context where the frequency and the risk are particularly high and higher than those reported in the studies cited above.

The high cesarean rate in sickle cell women is explained by the presence or conjunction of several risk factors: chronic fetal distress, vasculorenal syndrome, acute vasoocclusive crisis, or pelvic dystocia due to bone lesions of the pelvis [19]. The frequency found in our study is comparable to that of British, Indian, and Cuban studies where it varies from 53% to 72%, justified by fetal complications [9, 10, 14]. In sub-Saharan Africa, a comparable frequency (64%) is observed in Burkina-Faso [20], which is higher than those reported in Tanzania (26.8%) and Ghana (48, 7%) [3, 11]. In a previous study carried out in Brazzaville [7], it was most often prophylactic, motivated simply by the sickle cell field and without obstetric indication. The challenge in our context is to avoid systematizing this mode of delivery which, moreover, is associated with a high risk of vasoocclusive crisis in the postpartum [21]. As soon as childbirth is imminent, certain preventive measures, such as warming, oxygenation, and optimal hydration of the patient, even premature epidural locoregional analgesia, can be implemented to limit the risks of complications that can be induced by vaginal delivery [22].

Almost half of women with sickle cell disease experienced a vasoocclusive crisis in the postpartum period. The postpartum is a period at high risk of decompensation of sickle cell disease. Indeed, it combines maternal fatigue (working time, expulsive efforts), intense pain in the absence of epidural analgesia, fasting with dehydration, a state of metabolic acidosis linked to uterine muscle work and respiratory alkalosis (hyperventilation). All of these factors can lead to a vasoocclusive accident. Thus, the first 48 hours require close monitoring [22].

## 5. Conclusion

Maternal and fetal complications are significantly higher in pregnant sickle cell patients. A close supervisor of these pregnancies with a multidisciplinary approach between hematologist, obstetrician, and pediatrician is essential. Information, education, and communication sessions for sickle cell women are essential to minimize these risks.

## Figures and Tables

**Table 1:** Comparison of obstetric characteristics between women with SCD and non-SCD women seen in 4 maternity hospitals in Brazzaville between July 2017 and June 2019.

Variable	SCD (*n* = 65)	Non-SCD (*n* = 130)	OR	95% CI	*p*
Gravidity					
Median (*q*1–*q*3)	1 (0–2)	3 (1.5–4)			**0.0001**
Min–max	0–7	0–11			
Parity					
Median (*q*1–*q*3)	0.5 (0–1)	1 (0–3)			**0.0001**
Min–max	0–4	0–8			
History of miscarriage, *n* (%)	20 (30.7%)	71(54.6%)	**0.4**	**0.2–0.7**	**0.002**
History of fetal death in utero, *n* (%)	14 (21.6%)	6(4.6%)	**5.7**	**2.1–15.9**	**0.001**

SCD: sickle cell disease; non-SCD: non-sickle cell disease; CI: confidence interval; OR: odds ratio.

**Table 2:** Comparison of perinatal characteristics and the outcome of pregnancy between SCD women and non-SCD women seen in 4 maternity hospitals in Brazzaville between July 2017 and June 2019.

Outcomes	SCD	Non-SCD	*p*	OR	95% CI
*Bacterial infections*					
No	46 (70.7%)	124 (95.4%)			
Yes	19 (29.3%)	6 (4.6%)	0,001	21.7	7.6–62.7
Mean gestation age (weeks)	35	38			
Min–max	28–42	29–42	0,0001		
*Preterm birth*					
No	16 (24.6%)	90 (69.2%)			
Yes	49 (75.4%)	40 (30.8%)	0,001	8.0	3.0–23.2
*Mode of delivery*					
Vaginal	24 (37%)	86 (65.6%)			
Cesarean	41 (63%)	46 (35.4%)	0,001	3.1	1.6–5.7
Mean birth weight (g)	2400	3000			
Min–max	1400–3750	2675–4100	0,001		
*Low birth weight*					
No	31 (47.7%)	109 (83.9%)			
Yes	34 (52.3%)	21 (16.1%)	0,001	4.7	2.4–9.4
*NICU (n* *=* *188)*					
No	37 (59.7%)	104 (82.5%)			
Yes	25 (40.3%)	22 (17.5%)	0,01	3.2	1.6–6.3
*Fetal/child death (n* *=* *195)*					
No	51 (78.5%)	120 (93.2%)			
Yes	14 (21.5%)	10 (4.8%)	0,01	4.3	1.5–12.2
*Maternal outcome*					
Alive	61 (93.8%)	123 (94.6%)			
Died	4 (6.2%)	7 (5.4%)	0,9		

NICU: neonatal intensive care unit; SCD: sickle cell disease; non-SCD: non-sickle cell disease; CI: confidence interval; OR: odds ratio.

## Data Availability

The data used to support the findings of this study are included within the article.
